# Distinct nuclear orientation patterns for mouse chromosome 11 in normal B lymphocytes

**DOI:** 10.1186/1471-2121-15-22

**Published:** 2014-06-12

**Authors:** Ann-Kristin Schmälter, Alexandra Kuzyk, Christiaan H Righolt, Michaela Neusser, Ortrud K Steinlein, Stefan Müller, Sabine Mai

**Affiliations:** 1 Manitoba Institute of Cell Biology, University of Manitoba, Cancer Care Manitoba, 675 McDermot Avenue, Winnipeg, Manitoba, Canada; 2 Institute of Human Genetics, University Hospital, Ludwig-Maximilians-University, Goethestr. 29, 80336 Munich, Germany; 3 Department of Imaging Physics, Delft University of Technology, Delft, The Netherlands

**Keywords:** Chromosome orientation, Three-dimensional nucleus, Nuclear architecture, Fluorescence *in situ* hybridization, Multicolor banding, Chromosome territory

## Abstract

**Background:**

Characterizing the nuclear orientation of chromosomes in the three-dimensional (3D) nucleus by multicolor banding (mBANDing) is a new approach towards understanding nuclear organization of chromosome territories. An mBANDing paint is composed of multiple overlapping subchromosomal probes that represent different regions of a single chromosome. In this study, we used it for the analysis of chromosome orientation in 3D interphase nuclei. We determined whether the nuclear orientation of the two chromosome 11 homologs was random or preferential, and if it was conserved between diploid mouse Pre B lymphocytes of BALB/c origin and primary B lymphocytes of congenic [T38HxBALB/c]N wild-type mice. The chromosome orientation was assessed visually and through a semi-automated quantitative analysis of the radial and angular orientation patterns observed in both B cell types.

**Results:**

Our data indicate that there are different preferential patterns of chromosome 11 orientation, which are not significantly different between both mouse cell types (*p >* 0.05). In the most common case for both cell types, both copies of chromosome 11 were oriented in parallel with the nuclear border. The second most common pattern in both types of B lymphocytes was with one homolog of chromosome 11 positioned with its telomeric end towards the nuclear center and with its centromeric end towards the periphery, while the other chromosome 11 was found parallel with the nuclear border. In addition to these two most common orientations present in approximately 50% of nuclei from each cell type, other orientations were observed at lower frequencies.

**Conclusions:**

We conclude that there are probabilistic, non-random orientation patterns for mouse chromosome 11 in the mouse B lymphocytes we investigated (p < 0.0001).

## Background

Chromosomes occupy specific regions in the three-dimensional (3D) interphase nucleus, so-called chromosome territories (CTs) [1]. The radial arrangement of CTs shows cell-type specific differences [1,2]. The arrangement of CTs is influenced by many factors, such as chromosome size, gene density and transcription. In lymphocytes, chromosomes with a high gene-density are located further towards the center of the nucleus whereas chromosomes with a lower gene-density are concentrated at the nuclear periphery [1,3-6]. Transcription is also thought to play an important role in CT arrangement, with transcriptionally active genes usually located on the edge or outside of CTs and inactive genes found in the interior [7]. Gene expression can also cause chromatin movement in the 3D nucleus, as active genes may loop out of their CT altogether, presumably to access a transcription factory [8,9]. The correlation between the radial distribution of CTs and factors such as gene density, replication timing and transcription were examined by Küpper et al. [10]. They found that, in human cell nuclei, gene-density has a dominant impact on the radial distribution of CTs. In mouse cell nuclei other factors like guanine-cytosine content may, however, play a more important role in determining the radial distribution [11].

The position of each CT is established early in G1 and is maintained throughout interphase with minimal constrained diffusion [12]. Changes in the position of CTs have, however, been observed during cell differentiation, senescence and tumorigenesis. This occurs for example during adipocyte [13] and human epidermal keratinocyte differentiation [14]. In human fibroblasts, chromosome positions change when a cell becomes quiescent, senescent or when it re-enters the cell cycle [15,16].

In the present study, we investigated chromosome orientation for the first time in the mouse 3D nucleus. We used multicolor banding (mBANDing). A mBAND paint labels regions of a single chromosome with different fluorochromes. These different stains ensure that the centromeric end, telomeric end and interstitial regions can be differentiated from each other. In the 3D nucleus, the location of each region, and ultimately the orientation of the whole chromosome can, therefore, be determined. mBANDing is commonly used to study intrachromosomal changes in single chromosomes [17], but can also be applied in studies of nuclear architecture. Using mBAND probes, the degree of condensation of human chromosome 5 was determined in both interphase and metaphase [18], more recently the orientation of human chromosomes in sperm nuclei were analyzed [19]. In the latter study, the radial positions of all 24 CTs and their axial *vs*. non-axial as well as their linear *vs*. non-linear, orientations with respect to the sperm tail were determined, as well as the internal organization of chromosome subregions defined by different mBAND probes. A predominantly size-dependent radial arrangement was found for entire CTs. In addition, in particular for the smaller chromosomes, the authors also reported a gene density correlated orientation. Taken together, their study did not identify a preferential internal orientation of CTs with regard to the telomeric and centromeric end.

Our aim was to determine and compare the orientation of chromosome 11 in a diploid mouse PreB lymphocyte cell line and in primary B lymphocytes of congenic [T38HxBALB/c]N wild-type mice. Chromosome 11 is a gene dense chromosome [20]. The mBAND paint labels regions of chromosome 11 with four different fluorochromes. After fluorescence *in situ* hybridization (FISH) on 3D preserved cell nuclei, the location of centromeric, telomeric and interstitial regions and the orientation of chromosome 11 were visually determined for 300 nuclei per cell type. We observed three main patterns of chromosome 11 orientations. One arrangement involved chromosome 11 in parallel with the nuclear border, with neither the telomeric nor centromeric end pointing towards the nuclear center. Alternatively, the telomeric or the centromeric end of chromosome 11 were found pointing towards the nuclear center. Our data show that there is no significant difference between the frequencies of these three patterns of chromosome 11 orientations in both types of mouse B lymphocytes studied.

## Results

The mBANDing technique was used to study the nuclear organization of chromosome 11 in a diploid mouse Pre B lymphocyte line of BALB/c origin [21] and in B lymphocytes of congenic [T38HxBALB/c]N wild-type mice [22]. We visualized mBANDed chromosome 11 in metaphase preparations and chromosome territory (CT) 11 in 3D nuclei. Over 300 nuclei of both PreB and [T38HxBALB/c]N wild-type mouse lymphocytes were imaged using Axiovision 4.8 software (Carl Zeiss Inc. Canada). After deconvolution using a constrained iterative algorithm [23], all nuclei were analyzed by visual inspection to determine the orientation of both chromosome 11 homologs. To validate these results, we performed a semi-quantitative analysis of the radial arrangement of individual mBAND probe distributions on a subset of 45 nuclei per cell type using eADS software [10]. The 3D conformation of 90 individual chromosomes 11 from each of the two cell types was determined by measuring angles between the geometric centers of the different mBAND probes in individual chromosome territories. To determine whether cell cycle distribution had an impact on the chromosome 11 orientation patterns seen, cell cycle profiles of both B cell types were measured by FACS analysis.

### Chromosome 11 mBANDing in Pre B and T38H wt metaphase preparations

**Figure 1:**
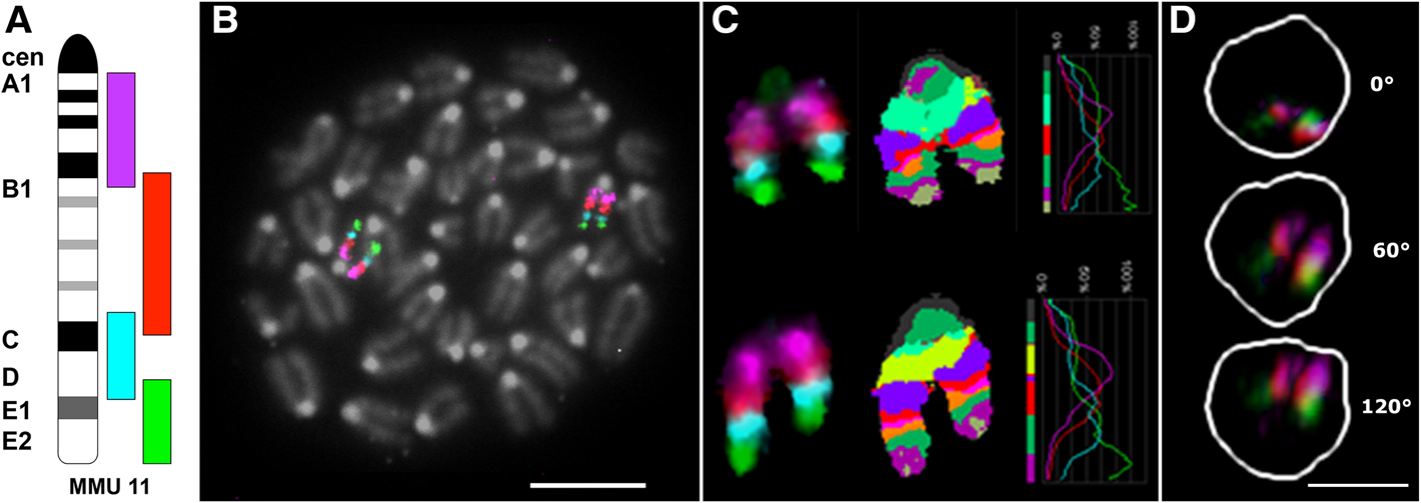
**mBAND labeling scheme, metaphase of PreB lymphocytes of BALB/c origin, mBANDed chromosomes 11 and representative [T38HxBALB/c]N wild-type B cell interphase nucleus. A** mBAND labeling scheme of mouse chromosome 11. Chromosome 11 is divided into four overlapping segments. Each segment was labeled with a different fluorochrome: the telomeric end in FITC (green), the centromeric end in Texas Red (magenta), and the interstitial segments in DEAC (cyan blue) and Gold (red), respectively. **B** Metaphase from diploid mouse Pre B lymphocytes of BALB/c origin after hybridization with the chromosome 11 mBAND probe. (Scale bar: 10 μm). **C** Two chromosome homologs from a different Pre B lymphocyte metaphase showing (from left to right) display colors, false colors and mBAND fluorescence intensity profiles. **D** Maximum intensity projections of a representative [T38HxBALB/c]N wild-type B cell interphase nucleus, from top to bottom: xy-view (z-projection), 60° and 120° rotation around the y-axis (note: the DEAC labeled probe is not shown because of insufficient signal/noise ratio of the FISH signal. The white line outlines the DAPI stained nucleus). (Scale bars are 5 μm).

Mouse chromosome 11 is approximately 122 Mb in size and has a mean gene density of 18.7 genes/Mb [24]. The mBAND probe set divides chromosome 11 into four overlapping segments, as illustrated in Figure 1A. The pericentromeric region is labeled with Texas Red, the proximal interstitial region in Gold, the distal interstitial region with DEAC and the telomeric region with FITC, respectively. Hybridization of the chromosome 11 mBAND probe to metaphase spreads (Figure 1B) was performed to examine copy number and structural stability of chromosome 11 in both cell types. Twenty metaphases in three independent FISH experiments were analyzed per cell type. We observed no structural or numerical aberrations in chromosome 11 in the cells studied. Figure 1B represents an example of a PreB lymphocyte metaphase, with both copies of chromosome 11 labeled by the mBAND probe. Figure 1C depicts the mBAND profiles of the two chromosomes from a PreB lymphocyte metaphase. All four fluorochromes can be clearly identified.

### mBANDing of chromosome 11 in interphase nuclei

We performed FISH on 3D preserved cell preparations from the two B cell types. With exception for the distal interstitial probe labeled with DEAC, all mBAND probes consistently showed the identification of specific FISH signals that were clearly distinguishable from non-specific background. We therefore excluded the DEAC labeled mBAND probe from all further measurements. Image z-stacks from 307 nuclei of PreB and 303 nuclei of [T38HxBALB/c]N wild-type mouse lymphocytes were captured and deconvolved (Materials and Methods). Figure 1D shows a representative chromosome 11 mBANDing image of a [T38HxBALB/c]N wild-type B lymphocyte 3D nucleus. 3D reconstructed images and movies representing various orientation patterns can be viewed in Additional files 1, 2, 3, 4, 5, 6, 7, 8, 9, 10, 11 and 12.

### Orientation of chromosome 11 in the 3D nucleus as examined by visual inspection

**Figure 2:**
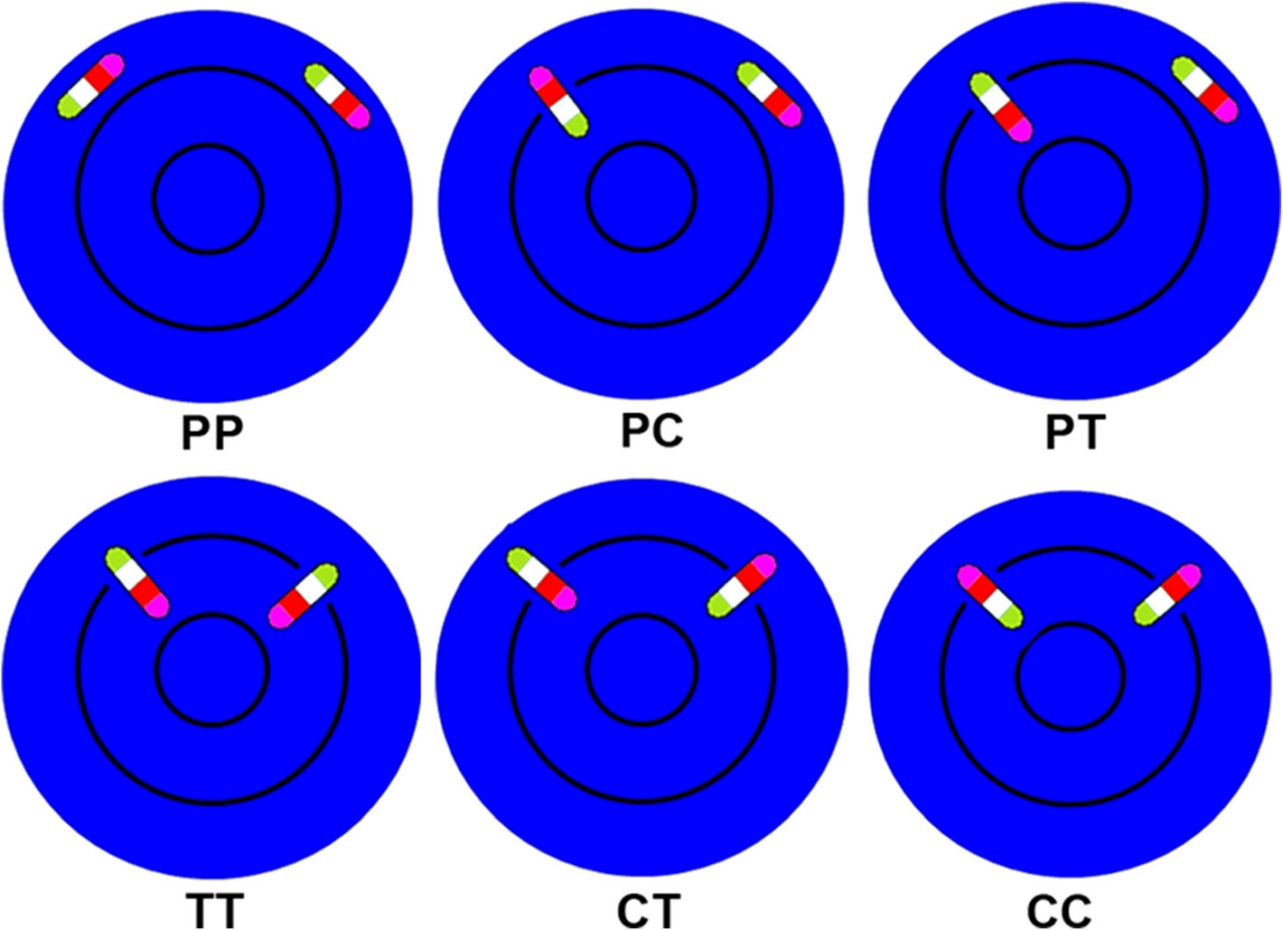
**Cartoon illustration of different possibilities of chromosome 11 orientation pairs in the nucleus.** The representative chromosome is composed of four differently colored segments; green segments represent telomeric ends, magenta segments centromeric ends, proximal and distal (not measured) interstitial segments are shown in cyan blue and red, respectively. The nucleus is illustrated in blue; the black circles divide the nucleus into central, intermediate and peripheral regions (PP = both homologs parallel to the nuclear periphery, PC/PT = one homolog is located in parallel to the periphery, while the other is oriented with its centromeric (red)/telomeric (green) end towards the nuclear periphery, TT = both homologs point with their telomeric (green) ends towards the periphery, CT = one homolog with its centromeric (red) end and the other homolog with its telomeric (green) end pointing towards the nuclear periphery, CC = both homologs point with their centromeric (red) ends towards the nuclear periphery, ). PP, PC and PT in the first row of Figure 2 are observed most frequently.

Using mBANDing, we were able to analyze the chromosome orientation of mouse chromosome 11 subregions for the first time in 3D interphase nuclei. We determined the frequency of nuclear chromosome orientation patterns by visual inspection in all captured nuclei from PreB lymphocytes and [T38HxBALB/c]N wild-type mice. mBAND territories were designated as parallel (“P”) to the nuclear surface when no chromosome end was pointing towards the nuclear center or the periphery. A homolog of chromosome 11 was classified as “C” when the centromeric region was localized closest to the nuclear border and “T when its telomeric end showed the most peripheral positioning. Figure 2 provides a cartoon illustration for the various orientation patterns observed. The most frequent pattern observed was with both copies of chromosome 11 located in parallel with the nuclear border (“PP”) (37.3% and 31.9%, respectively) (Table 1). There was no significant difference in the occurrence of this orientation pattern between both lymphocyte types against all other patterns combined (*p* = 0.20). The second most common orientation observed was with one homolog of chromosome 11 orientated with its telomeric end pointing towards the nuclear center, while the other chromosome 11 was parallel with the nuclear border (“PC”) (20.5% and 26.1% in PreB and [T38HxBALB/c]N wild-type mouse lymphocytes, respectively). There was no significant difference in the frequency of this orientation pattern seen between both types of lymphocytes (*p* = 0.13) (Table 1). One chromosome 11 pointing with its centromeric end to the center and the other in parallel with the nuclear border (“PT”) was the third most common orientation we observed. The occurrence of this orientation pattern did not differ significantly in both types of lymphocytes (*p* = 0.05) with a frequency of 16.3% of the PreB nuclei and of 10.4% of the [T38HxBALB/c]N wild-type lymphocyte nuclei (Table 1). In 10.7% of PreB and 5.8% of [T38HxBALB/c]N wild-type lymphocyte nuclei, both copies of chromosome 11 were orientated with their centromeric ends to the nuclear center (“TT”) (Table 1). Furthermore, in 9.1% in PreB and 7.3% [T38HxBALB/c]N wild-type lymphocyte nuclei one chromosome 11 pointed towards the nuclear center with its telomeric end and the other points towards the center with its centromeric end (“CT”) (Table 1). The scenario that both chromosomes 11 were orientated with their telomeric ends to the nuclear center (“CC”) was observed in 6.1% of the PreB and 18.5% of the [T38HxBALB/c]N wild-type mouse lymphocyte nuclei (Table 1).

**Table 1:** Frequencies of orientation patterns of chromosome 11

	**PreB [%]**	**[T38Hx BALB/c]N wt [%]**	**Chi-Square [p-value]**
**Both homologs in parallel to the nuclear border (PP)**	37.3	31.9	0.20
**One homolog points with its centromere to the nuclear periphery, the other is parallel to the nuclear border (PC)**	20.5	26.1	0.13
**One copy points with its telomere to the nuclear periphery, the other is parallel to the nuclear border (PT)**	16.3	10.4	0.05
**Both homologs point with their telomeric end to the nuclear periphery (TT)**	10.7	5.8	0.04
**One copy points with its telomeric end, and the other copy with centromeric end, to nuclear periphery (CT)**	9.1	7.3	0.45
**Both copies point with their centromeric ends to the nuclear periphery (CC)**	6.1	18.5	<0.0001

Orientation patterns of chromosome 11 and the frequency of orientation in Pre B lymphocytes of BALB/c origin and in B cells of congenic [T38HxBALB/c]N wild-type mice analyzed by visual inspection. A 2x2 Chi-Square test with p-value of >0.05 indicates that the frequency of the orientation is not significantly different between the two cell types. Each pattern was tested between the two cell types against all other patterns combined and the expected values were based on the marginal totals. There is a significant difference in the percentage of cells that display these major 2x3 orientation patterns between the two cell types (*p* <0.0001). (P = parallel, C = centromere points to periphery, T = telomere points to periphery).

By visual inspection we observed three main patterns of chromosome 11 orientation in the 3D interphase nucleus: (1) both homologs of chromosome 11 in parallel to the nuclear border (“PP”); (2) one copy of chromosome 11 in parallel to the border and the other copy pointing with its telomeric end towards the nuclear center (“PC”); (3) one copy of chromosome 11 in parallel to the border and the other copy pointing with its centromeric end towards the center (“PT”). Table 1 shows the frequencies of the different orientation patterns in the two cell types and 2x2 chi square *p*-values demonstrating the significant difference of each pattern with respect to all other orientation patterns combined. There was no significant difference in the occurrence of the orientation patterns observed between both B cell types (*p* > 0.05), with the exception of two minor patterns of orientation: when both copies of chromosome 11 pointed with their centromeric (“TT”) (p = 0.04) or telomeric (“CC”) (*p* < 0.0001) ends towards the nuclear center (Table 1).

A 2x3 contingency analysis of just the three major orientation patterns indicated that there was a significant difference in the distribution of the three patterns between the two cell type (*p* < 0.001).

### Semi-automated quantitative analysis of mBAND 11 orientation in mouse lymphocytes

To validate the above results, we analyzed nuclear orientation patterns of individual chromosomes 11 for a subset of 45 nuclei per cell type with the semi-automated software package eADS [9]. The mean radial arrangement of telomeric, interstitial and centromeric mBAND regions was determined in these 45 nuclei from both B cell types. We measured the 3D FISH signal distance to the nuclear surface in nm using eADS software [10], and then transformed these values to relative values (%) by normalizing it to the nuclear radius. Additional files 13, 14, 15 and Additional file 16: Table S1 show the results of the semi-automated software assisted analysis: The data indicate that this semi-quantitative analysis identifies, similar to the visual inspection, three most frequent orientation patterns for mouse chromosome 11.

When comparing the individual results of the quantitative analysis to the analysis by visual inspection of all 45 image per cell type we found, however, that only 30/45 (67%) nuclei of [T38HxBALB/c]N wild-type mouse lymphocyte and 28/45 (62%) nuclei of Pre B lymphocytes showed concordant results with both methods.

### 3D conformation of mouse chromosome 11

**Figure 3:**
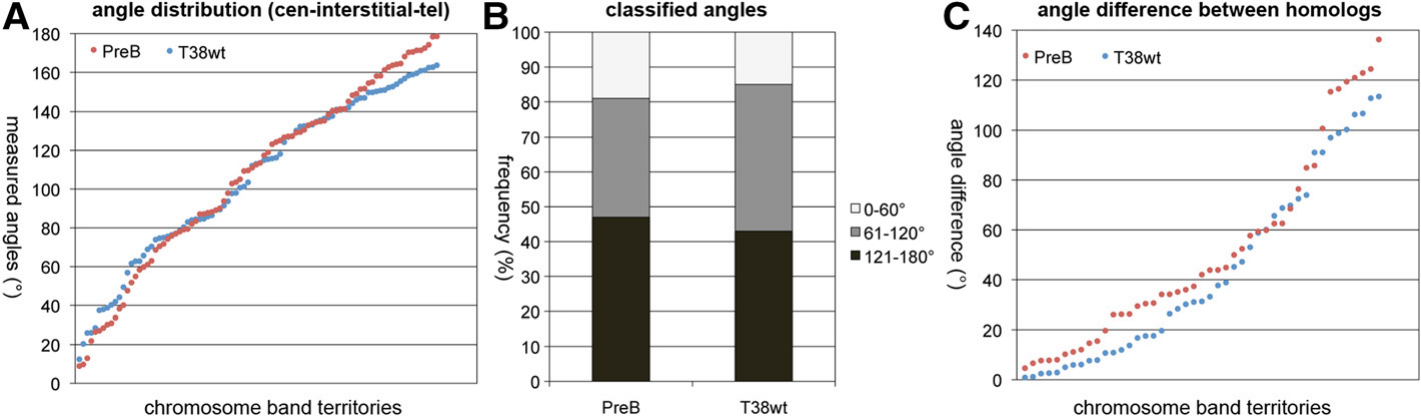
**3D conformation analysis of chromosome 11 mBAND territories in Pre B lymphocyte nuclei and in B cell nuclei of congenic [T38HxBALB/c]N wild-type mice using measured angles between geometric centers of centromeric, interstitial (apex) and telomeric chromosome band territories in individual chromosomes A Distribution of measured angles in each 90 PreB and [T38HxBALB/c]N wild-type B individual chromosomes 11. B** Bar diagram showing the percentage of angulated chromosomes with telomeric band territories in close proximity to centromeric regions (0-60° = chromosome 11 territory angulated), intermediate (61-120°) and straight chromosomes (121-180° = chromosome 11 territory straight) in the two cell lines. **C** Distribution of calculated angle differences between homologous CT pairs as a measure for the similarity of the 3D conformation of the two chromosomes 11 CT in individual nuclei.

We evaluated the 3D conformation of chromosome 11 in the two lymphocyte cell types using the software eADS [9]. We measured the angles between the geometric centers of centromeric, interstitial and telomeric mBAND territories using the interstitial segment as apex. Analysis of each 90 PreB and [T38HxBALB/c]N wild-type chromosomes using DistAngle software allowed us to determine whether the chromosome territory is preferentially straight or angulated during interphase. Mean measured angles in PreB cells (107°) were very similar compared to [T38HxBALB/c]N wildtype B cells (106°) and showed no statistically significant differences (*p* = 0.99). In both cell types we observed a broad distribution of measured angles (Figure 3A and 3B). 19% of PreB and 15% of [T38HxBALB/c]N wild-type B cell CT11 showed angles between 0 and 60° and were considered as rather angulated. 47% of PreB and 43% of [T38HxBALB/c]N wild-type B cell CT11 showed angles between 121° and 180° and indicate a trend towards a straight configuration (Figure 3A and B).

We also calculated the angle difference between homologous CT11 in individual nuclei. Again, we observed a broad distribution of measured angle differences in both B cell types ranging from 4°-136° (Figure 3C). Mean angle differences between homologs in PreB cells (51°) were slightly larger compared to [T38HxBALB/c]N wild-type B cells (43°), but no statistically significant differences were observed (*p* = 0.88).

### Cell cycle profiles

**Figure 4:**
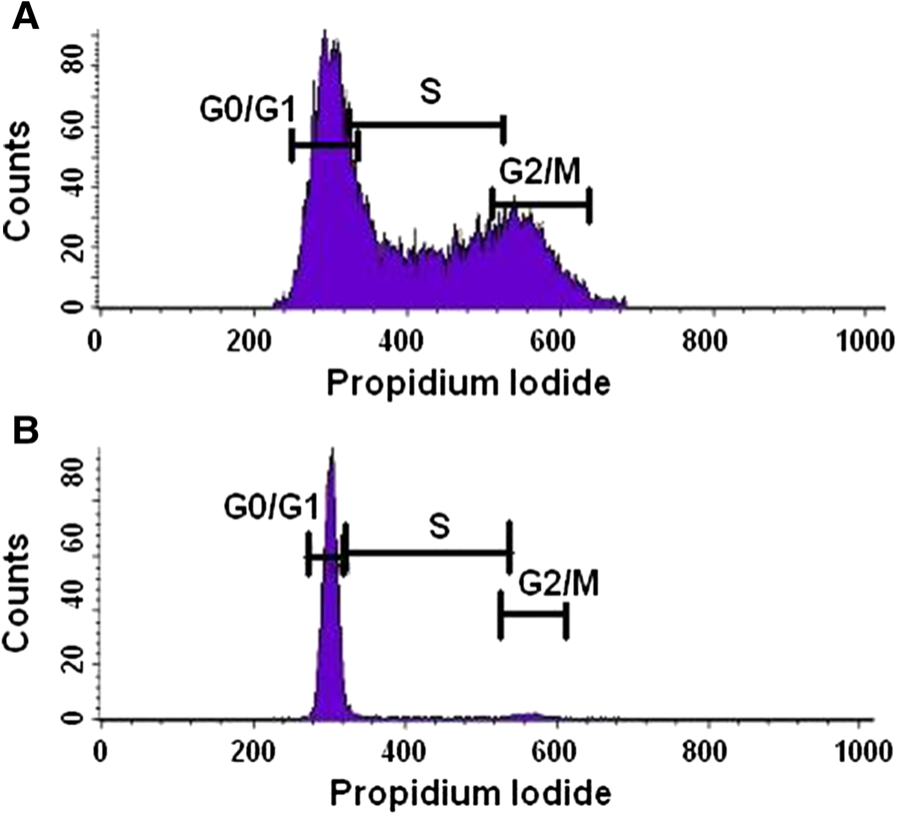
**Cell cycle profiles determined by FACS analysis**. The graphs illustrate the cell count in the different phases of the cell cycle, which correlates to the propidium iodide intensity. **A** The cell cycle profile of PreB lymphocytes of BALB/c origin. 45.08% of cells are found in G0/G1, 44.18% in S and 21.80% in G2/M. **B** The cell cycle profile of primary B cells from [T38HxBALB/c] wild-type mice. 94.76% of cells are found in G0/G1, 5.22% in s and 2.38% in G2/M.

To investigate whether cell cycle stages impact on the nuclear orientation and orientation patterns of mouse chromosome 11, we carried out fluorescent activated cell sorter (FACS) analysis of cell cycle profiles. The DNA profile of the PreB lymphocytes of BALB/c origin is shown in Figure 4A and the DNA profile of the [T38HxBALB/c]N wild-type mouse B cells is shown in Figure 4B. As the PreB lymphocytes were kept proliferating in culture, the cells are distributed throughout the different cell cycle phases with 45.08% in G0/G1, 44.18% in S and 21.80% in G2/M (Figure 4A). Contrastingly, the vast majority of the primary B cells are found in G0/G1 (94.76%) with only 5.22% in S and 2.38% in G2/M (Figure 4B). The comparison of the distinct cell cycle profiles observed for the [T38HxBALB/c]N wild-type mouse B cells and PreB cells indicates that the chromosome 11 orientation is independent of cell cycle profiles in the two B cell types examined in our study.

## Discussion

We used mBANDing for the first time to study chromosome orientation in the mouse 3D B cell nucleus. This technique has mostly been used previously to detect intrachromosomal rearrangements such as inversions, translocations and deletions in metaphase spreads [17]. One previous study used mBANDing to examine the spatial arrangements and the 3D configuration of CT in human sperm nuclei, demonstrating that mBANDing is an excellent technique to determine the 3D nuclear orientation of chromosomes [19]. Two alternative approaches were recently described, which could also be applied for a 3D topology analysis of mouse chromosomes, or could even be combined with the mouse mBAND probe set to further enhance the subregional resolution of the FISH banding probe set. One first approach used evolutionary rearranged chromosomes as subchromosomal probes for 3D-FISH [25], the second pooled BAC probes for predefined genomic regions or interest [26].

Using a mouse mBAND 11 probe set, we first determined chromosome 11 orientation by visual inspection. We then conducted a semi-automated quantitative analysis using the software eADS [9]. Both methods agreed on the identification and frequency of three different orientation patterns but did not coincide for each nucleus measured.

This result points to differences in the interpretation of mBAND data by the user (visual inspection) and by the user performing semi-automated analysis that includes the manual segmentation of the regions of interest. During this procedure, some pixels might have been discarded or identified as artifacts when cutting out the bands separately in different channels with no overview of the whole chromosome. It was not possible to distinguish low grey levels from each other or background pixels with value 0, which may lead to mistakes in the manual segmentation. The pixels of the bands were measured with respect to the nucleus stained in DAPI. If DAPI staining was weaker in some cells than in others, then the subsequent identification of the positions of the band pixels are measured inaccurately. Due to the lack of a fully automated program, these current measurements are only indicative of varied chromosome orientation patterns in the cells examined, but no absolute measure of chromosome band positioning. We are currently working towards a fully automated program.The PreB cells were kept proliferating in culture, while the majority (94.76%) of the primary B cells were directly isolated from the mice (kept under specific pathogen free (SPF) conditions) and are in G0/G1 of the cell cycle; this was confirmed through FACS cell cycle analysis (Figure 4). In cycling PreB cells no correlation was found between cell cycle stage and radial orientation of mBAND CT. We have not determined the cell cycle stage of the individual nuclei, e.g. by BrdU, so we can only state “no difference between cycling and quiescent cells” The influences of differentiation and gene density on the nuclear architecture and on the radial orientation of gene dense telomeric chromatin appear to be a more likely cause for the observed differences between the two cell types. Gene density can be ruled out as cause for differences because it is the same in both cell types. This leaves differentiation as cause, which is linked to transcription.

Transcription may influence chromosome position. Regions of chromatin have been shown to change position in the nucleus with activation and increased levels of transcription [8,27,28]. Transcription factories can also alter the arrangement of chromatin as genes may loop out of their territory to be transcribed [29] or multiple genes will meet to be co-transcribed in a single transcription factory [30]. The access to transcription factories , therefore, may be relevant for the orientation of chromosomes in the nucleus. A possible rotation of a chromosome in the nucleus may be an alternate way in which active regions can access the transcription machinery. For example, chromosome rotation could occur around the centromeric or telomeric end. This would allow genes that lay close to rotation axis to remain in a stable position whereas those further away will undergo a positional change. Alternatively, or in addition to rotation, a chromosome territory may adopt different large-scale folding patterns in order to reach this goal, which may result in straight or angulated 3D conformations. Additionally, the active state of chromatin with regards to methylation, acetylation and other post-translational histone modifications, may not only alter chromatin condensation but also orientation. To test for the effects of transcription on chromosome orientation was beyond the scope of this study.

In approximately 45% of the nuclei examined, it was observed that the two homologs of chromosome 11 did not have the same orientation. This may be an innate protection mechanism of the cell. It has been shown by Heride et al. [31] that chromosomes are non-randomly closer to a heterologue than a homologue. This led them to propose that it may be evolutionarily important to position chromosomes in this way to avoid homologous recombination and to avoid damaging both copies of a chromosome by a single genotoxic stress event. The same argument may explain why two copies of a chromosome may adopt different orientations in the nucleus.

In this study we examined, for the first time, chromosome orientation in the murine 3D nucleus using mBANDing. We found that the orientation of chromosome 11 in PreB lymphocytes of BALB/c origin and primary B cells of congenic [T38HxBALB/c]N wild-type mice varies between distinct patterns of orientation. Gene density has been thought to be the primary factor influencing lymphocyte CT arrangement. We find, however, that it is not sufficient to determine the orientation of mouse chromosome 11 in B cells. Because gene-density is stable, it cannot account for differences in the three major patterns of chromosome 11 orientation we observed. Although we did not find orientation pattern and gene-density to be linked for chromosome 11 in both B-cell models, this may not hold true for all mouse chromosomes and cell types and should be assessed for other chromosomes and non B lineage cell types. At present, our findings allow us to conclude that chromosomes may display distinct orientations in the interphase nucleus of diploid mouse B cells during some stages of differentiation, but the underlying mechanisms require further investigation.

## Conclusion

We analyzed for the first time the orientation of mouse chromosome 11 with respect to the telomeric and centromeric ends of chromosomes in the 3D interphase nucleus. Distinct orientation patterns of CT 11 in PreB lymphocytes of BALB/c origin and primary B cells of congenic [T38HxBALB/c]N wild-type mice were observed. The most frequent pattern was with both homologs positioned in parallel to the nuclear border in both types of B lymphocytes. Alternatively the telomeric or centromeric end was found pointing towards the nuclear periphery or center. Overall, chromosome orientation appears to be a non-random feature of the genome in interphase nuclei.

## Methods

### Cell harvest and cell culture

Mouse PreB lymphocytes of BALB/c origin [21] were cultured in RPMI 1640 media supplemented with 10% heat-inactivated fetal bovine serum (FBS), 1% penicillin-streptomycin, 1% L-Glutamine, 1% sodium pyruvate and 0.1% β-mercaptoethanol (Invitrogen/Gibco, Burlington, ON, Canada). Cells were incubated at 37°C.

Primary B cells were harvested from spleens of three 6 to 8 weeks old congenic [T38HxBALB/c]N wild-type mice [22]. Procedures were conducted according to Animal Protocol 11-019, approved by Central Animal Care Services, University of Manitoba (Winnipeg, MB, Canada).

### 2D and 3D fixation

2D chromosome fixation was conducted as described by Mai and Wiener [32]. Primary B cells and PreB lymphocytes were centrifuged for 10 minutes at 1000 rpm. The pellet was resuspended in 0.075 M KCl for 30 minutes. Next the cells underwent centrifugation for 10 minutes at 1000 rpm and then a drop fixation with 3:1 methanol to acetic acid. After resuspension of the pellet, cells were dropped onto slides.

3D nuclei fixation was conducted as described by Solovei et al. [33]. Primary B cells and PreB lymphocytes were centrifuged for 10 minutes at 1000 rpm. After resuspension of the pellet, cells were applied to slides. One hour later, the slides were washed in 1 × phosphate-buffered saline (PBS), 0.3xPBS and then incubated in freshly prepared 3.7% formaldehyde. Next the slides were washed in 0.05% Triton-X-100/1xPBS, followed by a wash in 0.5% Triton-X-100/1xPBS. The slides were then incubated in 20% Glycerol/1×PBS, for at least an hour, and subsequently underwent repeated freeze/thaw cycles in liquid nitrogen. Afterwards, the slides were washed in 0.05% Triton-X-100/1xPBS, followed by incubation in 0.1 M HCl. After washing the slides in 2× saline sodium citrate buffer (SSC) they were kept at least one hour in 50% formamide/2×SSC at 4°C.

### Fluorescence-activated cell sorting (FACS) analysis

FACS analysis was conducted as described by Caporali et al. [34]. Briefly, PreB lymphocytes BALB/c origin and primary B cells of [T38HxBALB/c]N wild-type mice were fixed in 70% cold ethanol and incubated at 4°C overnight. The following day, the pellet was washed twice with 1% FBS in 1xPBS after centrifugation at 1200 rpm for ten minutes. The final pellet was resuspended in 1×PBS and stained with propidium iodide (1 μg/mL) (Sigma Aldrich, Oakville, ON, Canada). Flow cytometry was used to analyze the cell cycle profiles using a FACSCalibur (Becton Dickinson, Mississauga, ON, Canada).

### Multicolor banding

The mBANDing probe for mouse chromosome 11 was previously described by Benedek et al. [35] and was purchased from Metasystems (Altussheim, Germany) for the present experiments. The pericentromeric region is labeled with Texas Red, the proximal interstitial region in Gold, the distal interstitial region in DEAC and the telomeric region with FITC, respectively. First, slides were equilibrated in 2×SSC, followed by an RNAase A treatment (100 μg/ml) in 2×SSC for one hour at 37°C, and then incubation in freshly prepared 0.01 M HCl with 100 μg/ml pepsin for two minutes. After washing the slides in 1xPBS, they were pretreated in 1% formaldehyde in 1×PBS/50 mM MgCl2, followed by a wash in 1×PBS. For denaturation, the slides were incubated in 0.1×SSC, and then transferred into 2×SSC at 70°C for 30 minutes. After the solution was cooled down to 37°C, the slides were transferred to 0.1×SSC and then denatured in 0.07 M NaOH at room temperature for one minute. Before dehydration in ethanol (30%, 50%, 70% and 90%), the slides were placed in 0.1×SSC and then 2×SSC at 4°C. Next, the mBANDing probe was applied as recommended, sealed to the slide with rubber cement and incubated for two days at 37°C. Post-hybridization washes included 1×SSC at 75°C and in 4×SSC/0.05% Tween20. The chromatin was counterstained with 4’6’-diamidino-2-phenylindole (DAPI) and mounted with ProLong Gold antifade (Invitrogen/Gibco, Burlington, ON, Canada).

### Image acquisition

Two-dimensional image acquisition was performed using an Axioplan 2 microscope (Carl Zeiss Ltd., Toronto, ON, Canada) with a 63x/1.4 oil objective lens (Carl Zeiss Ltd., Toronto, ON, Canada) and the ISIS-FISH imaging system 5.0 SR 3 (Metasystems Group Inc. Boston, MA, USA). A DAPI filter was used to visualize the chromosomal counterstain. To detect the four regions of chromosome 11 that were labeled with different fluorochromes (DEAC, FITC, Gold and TexasRed, respectively), appropriate narrow band pass filters were used (Chroma Technologies). The region pseudo-colored in green was detected by a SP-101 fluorescein isothiocyanate (FITC) filter (Excitation CWL/Bandwidth: 471 nm/39 nm, Emission CWL/Bandwidth: 522 nm/40 nm, Chroma Series No.: SP100), the region pseudo-colored in cyan by a 31036v2 7-diethylaminocoumarin-3-carboxylic acid (DEAC) filter (Excitation CWL/Bandwidth: 436 nm/20 nm, Emission CWL/Bandwidth: 480 nm/30 nm, Chroma Series No.: 31000 Series), the region pseudo-colored in red by a 11006v3 Gold filter (Excitation CWL/Bandwidth: 350 nm/50 nm, Emission CWL/Bandwidth: 515 nm/nm, Chroma Series No.: 11000 Series) and the region pseudo-colored in magenta by a 41004 Texas Red® filter (Excitation CWL/Bandwidth: 560 nm/55 nm, Emission CWL/Bandwidth: 645 nm/75 nm, Chroma Series No.: 41000 Series).

For the 3D image acquisition, an AxioImager Z2 microscope (Carl Zeiss Inc. Canada) equipped with the same filter sets as for 2D image acquisition and an AxioCam MRm (Carl Zeiss Inc. Canada) was used, combined with Axiovision 4.8 software (Carl Zeiss Inc. Canada). To reconstruct a 3D image, z-stacks of 80 slices, with 200 nm axial distance and 102 nm x/y pixel size were acquired. Deconvolution was performed with a constrained iterative algorithm [23] using Axiovision 4.8 software (Carl Zeiss Inc. Canada). The chromosome orientation was determined by visual inspection based on the mBAND FISH pattern.

### Quantitative semi-automated analysis

Quantitative measurements of mean radial mBAND probe distributions with respect to the nuclear border were performed using the software eADS, a 3D distance measurement tool described in detail by Küpper et al. [10]. In short, the pixels are manually classified as being band or not and being nucleus or not. The Euclidean distance between the nuclear surface and each bands pixel is then measured from these images. For the probe distributions each pixel in each band is used, for the determination of the orientation only the smallest distance for each band is used. The orientation was determined by the difference in radial position of the telomeric and centromeric band. When the difference was smaller than N percentpoints it was called parallel. The software DistAngle [36] was employed to measure 3D angles between geometric centers of different mBAND probes from individual chromosomes.

### Statistical analysis

The different orientations of chromosome 11 were compared to each other and compared between the different types of lymphocytes using all of the following tests: Chi-Square, Likelihood Ratio Chi-Square, Continuity Adj Chi-Square and Mantel Haenszel Chi-Square analysis. The *p*-values of test results shown here for the comparison of different orientations are the Chi-Square values. All other tests yielded similar results (data not shown).

The Mann-Whitney rank sum test was used to determine statistically significant differences in the median radial arrangement of mBAND FISH signals. A *p*-value <0.05 was considered significant.

## Abbreviations

2D: Two-dimensional; 3D: Three-dimensional; CT: Chromosome territory; DAPI: 4’6’-diamidino-2-phenylindole; DEAC: 7-diethylaminocoumarin-3-carboxylic acid; FACS: Fluorescence-activated cell sorting; FISH: Fluorescent *in situ* hybridization; FITC: Fluorescein isothiocyanate; FBS: Fetal bovine serum; mBANDing: Multicolor banding; PBS: Phosphate-buffered saline; SSC: Saline sodium citrate buffer.

## Competing interests

The authors declare that they have no competing interests.

## Author’s contribution

AKS carried out 2D and 3D cell preparation and fixation, mBANDing, image acquisition, visual inspection, conducted the analysis by the software eADs and drafted the manuscript. AK contributed to FACS, contributed to 2D and 3D fixation of cells and to the writing of the manuscript. CR contributed to the discussion, created 3D movies, assisted in data analysis and contributed to writing the manuscript. MN participated in the analysis by eADS. OS is AKS’ co-supervisor. SMü conducted the analysis by eADS and contributed to writing the paper. SMa designed the study and helped to draft the manuscript. All authors read and approved the final manuscript.

## Supplementary Material

Additional file 1 3D movie of a [T38HxBALB/c]N wild type B cell nucleus with the orientation pattern “CP”. Click here for file

Additional file 2 3D movie illustrating a Pre B nucleus with the orientation pattern “PP”. Click here for file

Additional file 3 **3D view of a Pre B nucleus with the orientation pattern “CT”.** One chromosome 11 is oriented with its centromeric end (red) towards the nuclear center, whereas the other chromosome 11 is oriented with its telomeric end (green) towards the center. Click here for file

Additional file 4 3D movie illustrating a Pre B nucleus with the orientation pattern “TP”. Click here for file

Additional file 5 3D view of a Pre B nucleus with the orientation pattern “PP”. Both copies of chromosome 11 are located in parallel to the nuclear periphery. Click here for file

Additional file 6 3D movie illustrating a Pre B nucleus with the orientation pattern “TT”. Click here for file

Additional file 7 **3D view of a Pre B nucleus with the orientation pattern “TP”.** One chromosome 11 is oriented with its centromeric end (red) towards the nuclear center and with its telomeric end (green) towards the periphery, the other chromosome is located in parallel to the nuclear periphery. Click here for file

Additional file 8 3D movie illustrating a Pre B nucleus with the orientation pattern “CC”. Click here for file

Additional file 9 **3D view of a Pre B nucleus with the orientation pattern “TT”.** Both copies of chromosome 11 are pointing with their centromeric ends (red) towards the nuclear center, whereas their telomeric ends (green) are pointing towards the periphery. Click here for file

Additional file 10 **3D view of a [T38HxBALB/c]N wild type B cell nucleus with the orientation pattern “CP”.** One chromosome 11 is located in parallel to the nuclear periphery, the other is oriented with its telomeric end (green) towards the center. Click here for file

Additional file 11 3D movie of a [T38HxBALB/c]N wild type B cell nucleus with the orientation pattern “CP”. Click here for file

Additional file 12 **3D view of a [T38HxBALB/c]N wild type B cell nucleus with the orientation pattern “CC”.** Both copies of chromosome 11 are pointing with their telomeric ends (green) towards the nuclear center. Click here for file

Additional file 13 **Mean radial distribution of the centromeric, interstitial and telomeric mBAND FISH signals in A PreB and B T38wt cell nuclei.** The 3D FISH signal distance to the nuclear surface was measured nm using eADS software (Küpper et al., 2007) (n = number of nuclei, nm = nanometer). **A** In PreB cells the mean radial position of the telomeric segment was at 2016 nm and 42% relative distance from the nuclear surface, the interstitial segment at 2178 nm (46%) and the centromeric segment at 2151 nm (45%). **B** In T38wt cells mean absolute and relative probe distances to nuclear surface were 1906 nm (56%) for the telomeric region, 1617 nm (47%) for the interstitial and 1522 nm (44%) for the centromeric region. A small percentage of nuclei shows band signals beyond the nuclear border. This is probably due to weak nuclear DAPI stain measured by the software eADS (see Discussion). Click here for file

Additional file 14 **Normalized (% distance to nuclear surface) radial centromeric, interstitial and telomeric mBAND probe distributions in individual chromosomes from each 45 A PreB and B [T38HxBALB/c]N wild-type B cell nuclei using eADS software.** The 3D FISH signal distance to the nuclear surface in nm was transformed to relative values (%) by normalization using the nuclear radius as reference. Each colored dot represents the radial position of the geometric center from an individual chromosome 11. The two chromosome 11 homologs from each nucleus are shown side by side. Nuclei with a similar radial orientation of the two homologs are depicted in clusters separated by bold vertical lines. mBAND territories were designated as parallel (“P”) to the nuclear surface when each of the measured relative radial distances between centromeric, telomeric and interstitial chromosome segments was less than 15% of the nuclear radius. This equals to approximately a 400-500 nm radial distance depending on the size of the respective nucleus. We chose a 15% cut-off level because this is approximately twice the distance between consecutive image z-sections of 200 nm. Consequently, in chromosome 11 territories where at least one of the measured relative distances between mBAND territories would exceed 15% the CT was assigned an orientation with either the telomeric or the interstitial or the centromeric end pointing towards the nuclear periphery or center. (P = parallel, C = centromere points to periphery, I = interstitial is most peripheric, T = telomere points to periphery). Click here for file

Additional file 15 **Relative radial orientation of 45 homologous chromosome 11 mBAND CT pairs from each 45 PreB and [T38HxBALB/c]N wildtype B cell interphase nuclei. Frequencies (%) of homologous CT pairs showing different combinations of radial orientations.** (P = parallel, C = centromere points to periphery, I = interstitial is most peripheric, T = telomere points to periphery). Click here for file

Additional file 16: Table S1 Results of semi-automated quantitative analysis using the software eADS. Frequencies of relative orientation patterns of homologous chromosomes in individual nuclei. (P=parallel, C=centromere points to periphery, I=interstitial is most peripheric, T=telomere points to periphery). Click here for file

## References

[B1] Cremer T Cremer M Chromosome territories Cold Spring Harb Perspect Biol 2010 2 3 a003889 20300217 10.1101/cshperspect.a003889 PMC2829961

[B2] Bolzer A Kreth G Solovei I Koehler D Saracoglu K Fauth C Muller S Eils R Cremer C Speicher MR Cremer T Three-dimensional maps of all chromosomes in human male fibroblast nuclei and prometaphase rosettes PLoS Biol 2005 3 5 e157 15839726 10.1371/journal.pbio.0030157 PMC1084335

[B3] Croft JA Bridger JM Boyle S Perry P Teague P Bickmore WA Differences in the localization and morphology of chromosomes in the human nucleus J Cell Biol 1999 145 6 1119 1131 10366586 10.1083/jcb.145.6.1119 PMC2133153

[B4] Boyle S Gilchrist S Bridger JM Mahy NL Ellis JA Bickmore WA The spatial organization of human chromosomes within the nuclei of normal and emerin-mutant cells Hum Mol Genet 2001 10 3 211 219 11159939 10.1093/hmg/10.3.211

[B5] Tanabe H Habermann FA Solovei I Cremer M Cremer T Non-random radial arrangements of interphase chromosome territories: evolutionary considerations and functional implications Mutat Res 2002 504 1–2 37 45 12106644 10.1016/s0027-5107(02)00077-5

[B6] Saccone S Federico C Bernardi G Localization of the gene-richest and the gene-poorest isochores in the interphase nuclei of mammals and birds Gene 2002 300 1–2 169 178 12468098 10.1016/s0378-1119(02)01038-7

[B7] Morey C Kress C Bickmore WA Lack of bystander activation shows that localization exterior to chromosome territories is not sufficient to up-regulate gene expression Genome Res 2009 19 7 1184 1194 19389823 10.1101/gr.089045.108 PMC2704431

[B8] Lanctot C Cheutin T Cremer M Cavalli G Cremer T Dynamic genome architecture in the nuclear space: regulation of gene expression in three dimensions Nat Rev Genet 2007 8 2 104 115 17230197 10.1038/nrg2041

[B9] Sutherland H Bickmore WA Transcription factories: gene expression in unions? Nat Rev Genet 2009 10 7 457 466 19506577 10.1038/nrg2592

[B10] Kupper K Kolbl A Biener D Dittrich S von Hase J Thormeyer T Fiegler H Carter NP Speicher MR Cremer T Cremer M Radial chromatin positioning is shaped by local gene density, not by gene expression Chromosoma 2007 116 3 285 306 17333233 10.1007/s00412-007-0098-4 PMC2688818

[B11] Hepperger C Mannes A Merz J Peters J Dietzel S Three-dimensional positioning of genes in mouse cell nuclei Chromosoma 2008 117 6 535 551 18597102 10.1007/s00412-008-0168-2

[B12] Thomson I Gilchrist S Bickmore WA Chubb JR The radial positioning of chromatin is not inherited through mitosis but is established de novo in early G1 Curr Biol 2004 14 2 166 172 14738741 10.1016/j.cub.2003.12.024

[B13] Kuroda M Tanabe H Yoshida K Oikawa K Saito A Kiyuna T Mizusawa H Mukai K Alteration of chromosome positioning during adipocyte differentiation J Cell Sci 2004 117 Pt 24 5897 5903 15537832 10.1242/jcs.01508

[B14] Marella NV Seifert B Nagarajan P Sinha S Berezney R Chromosomal rearrangements during human epidermal keratinocyte differentiation J Cell Physiol 2009 221 1 139 146 19626667 10.1002/jcp.21855

[B15] Bridger JM Boyle S Kill IR Bickmore WA Re-modelling of nuclear architecture in quiescent and senescent human fibroblasts Curr Biol 2000 10 3 149 152 10679329 10.1016/s0960-9822(00)00312-2

[B16] Mehta IS Amira M Harvey AJ Bridger JM Rapid chromosome territory relocation by nuclear motor activity in response to serum removal in primary human fibroblasts Genome Biol 2010 11 1 R5 20070886 10.1186/gb-2010-11-1-r5 PMC2847717

[B17] Chudoba I Plesch A Lorch T Lemke J Claussen U Senger G High resolution multicolor-banding: a new technique for refined FISH analysis of human chromosomes Cytogenet Cell Genet 1999 84 3–4 156 160 10393418 10.1159/000015245

[B18] Lemke J Claussen J Michel S Chudoba I Muhlig P Westermann M Sperling K Rubtsov N Grummt UW Ullmann P Kromeyer-Hauschild K Liehr T Claussen U The DNA-based structure of human chromosome 5 in interphase Am J Hum Genet 2002 71 5 1051 1059 12370837 10.1086/344286 PMC385084

[B19] Manvelyan M Hunstig F Bhatt S Mrasek K Pellestor F Weise A Simonyan I Aroutiounian R Liehr T Chromosome distribution in human sperm - a 3D multicolor banding-study Mol Cytogenet 2008 1 25 19014589 10.1186/1755-8166-1-25 PMC2613144

[B20] Sakharkar MK Perumal BS Sakharkar KR Kangueane P An analysis on gene architecture in human and mouse genomes In Silico Biol 2005 5 4 347 365 16268780

[B21] Mai S Hanley-Hyde J Rainey GJ Kuschak TI Paul JT Littlewood TD Mischak H Stevens LM Henderson DW Mushinski JF Chromosomal and extrachromosomal instability of the cyclin D2 gene is induced by Myc overexpression Neoplasia 1999 1 3 241 252 10935479 10.1038/sj.neo.7900030 PMC1508077

[B22] Wiener F Schmalter AK Mowat MR Mai S Duplication of Subcytoband 11E2 of chromosome 11 is regularly associated with accelerated tumor development in v-abl/myc-Induced Mouse Plasmacytomas Genes Cancer 2010 1 8 847 858 21779468 10.1177/1947601910382897 PMC3092250

[B23] Schaefer LH Schuster D Herz H Generalized approach for accelerated maximum likelihood based image restoration applied to three-dimensional fluorescence microscopy J Microsc 2001 204 Pt 2 99 107 11737543 10.1046/j.1365-2818.2001.00949.x

[B24] Genome reference consortium mouse build 38 http://www.ncbi.nlm.nih.gov/assembly/GCF_000001635.20/

[B25] Neusser M Schubel V Koch A Cremer T Muller S Evolutionarily conserved, cell type and species-specific higher order chromatin arrangements in interphase nuclei of primates Chromosoma 2007 116 3 307 320 17318634 10.1007/s00412-007-0099-3

[B26] Teller K Illner D Thamm S Casas-Delucchi CS Versteeg R Indemans M Cremer T Cremer M A top-down analysis of Xa- and Xi-territories reveals differences of higher order structure at >/=20 Mb genomic length scales Nucleus 2011 2 5 465 477 21970989 10.4161/nucl.2.5.17862

[B27] Chuang CH Carpenter AE Fuchsova B Johnson T de Lanerolle P Belmont AS Long-range directional movement of an interphase chromosome site Curr Biol 2006 16 8 825 831 16631592 10.1016/j.cub.2006.03.059

[B28] Akhtar A Gasser SM The nuclear envelope and transcriptional control Nat Rev Genet 2007 8 7 507 517 17549064 10.1038/nrg2122

[B29] Volpi EV Chevret E Jones T Vatcheva R Williamson J Beck S Campbell RD Goldsworthy M Powis SH Ragoussis J Trowsdale J Sheer D Large-scale chromatin organization of the major histocompatibility complex and other regions of human chromosome 6 and its response to interferon in interphase nuclei J Cell Sci 2000 113 Pt 9 1565 1576 10751148 10.1242/jcs.113.9.1565

[B30] Osborne CS Chakalova L Mitchell JA Horton A Wood AL Bolland DJ Corcoran AE Fraser P Myc dynamically and preferentially relocates to a transcription factory occupied by Igh PLoS Biol 2007 5 8 e192 17622196 10.1371/journal.pbio.0050192 PMC1945077

[B31] Heride C Ricoul M Kieu K von Hase J Guillemot V Cremer C Dubrana K Sabatier L Distance between homologous chromosomes results from chromosome positioning constraints J Cell Sci 2010 123 Pt 23 4063 4075 21084563 10.1242/jcs.066498

[B32] Mai S Wiener F The impact of p53 loss on murine plasmacytoma development Chromosome Res 2002 10 3 239 251 12067213 10.1023/a:1015200307448

[B33] Solovei I Cavallo A Schermelleh L Jaunin F Scasselati C Cmarko D Cremer C Fakan S Cremer T Spatial preservation of nuclear chromatin architecture during three-dimensional fluorescence in situ hybridization (3D-FISH) Exp Cell Res 2002 276 1 10 23 11978004 10.1006/excr.2002.5513

[B34] Caporali A Wark L Vermolen BJ Garini Y Mai S Telomeric aggregates and end-to-end chromosomal fusions require myc box II Oncogene 2007 26 10 1398 1406 16953226 10.1038/sj.onc.1209928

[B35] Benedek K Chudoba I Klein G Wiener F Mai S Rearrangements of the telomeric region of mouse chromosome 11 in Pre-B ABL/MYC cells revealed by mBANDing, spectral karyotyping, and fluorescence in-situ hybridization with a subtelomeric probe Chromosome Res 2004 12 8 777 785 15702416 10.1007/s10577-005-5264-z

[B36] Grasser F Neusser M Fiegler H Thormeyer T Cremer M Carter NP Cremer T Muller S Replication-timing-correlated spatial chromatin arrangements in cancer and in primate interphase nuclei J Cell Sci 2008 121 Pt 11 1876 1886 18477608 10.1242/jcs.026989 PMC2687722

