# Supplementary figures and images for: Distinct nuclear orientation patterns for mouse chromosome 11 in normal B lymphocytes
Source: BMC Cell Biol. 2014 Jun 12;15:22. doi: 10.1186/1471-2121-15-22 (PMC4078936; doi:10.1186/1471-2121-15-22)

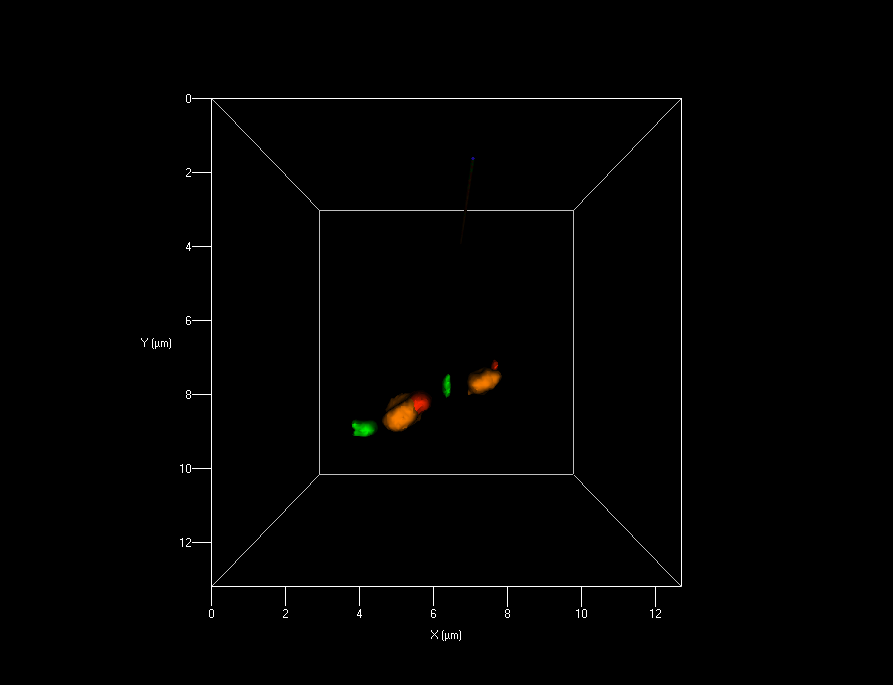

Supplement: Additional file 3 — 3D view of a Pre B nucleus with the orientation pattern “CT”. One chromosome 11 is oriented with its centromeric end (red) towards the nuclear center, whereas the other chromosome 11 is oriented with its telomeric end (green) towards the center. [file 1471-2121-15-22-S3.tiff]

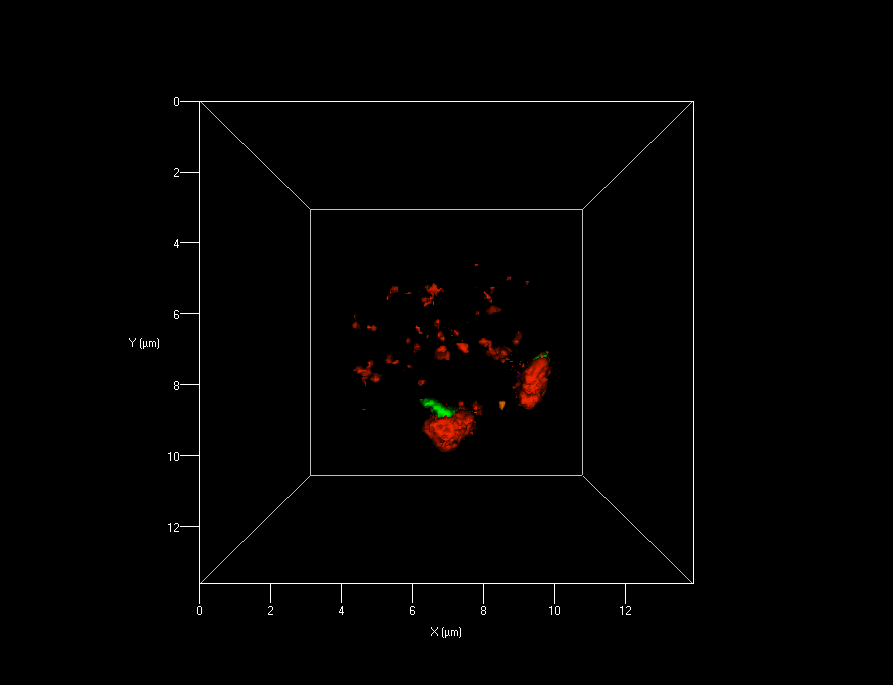

Supplement: Additional file 5 — 3D view of a Pre B nucleus with the orientation pattern “PP”. Both copies of chromosome 11 are located in parallel to the nuclear periphery. [file 1471-2121-15-22-S5.tiff]

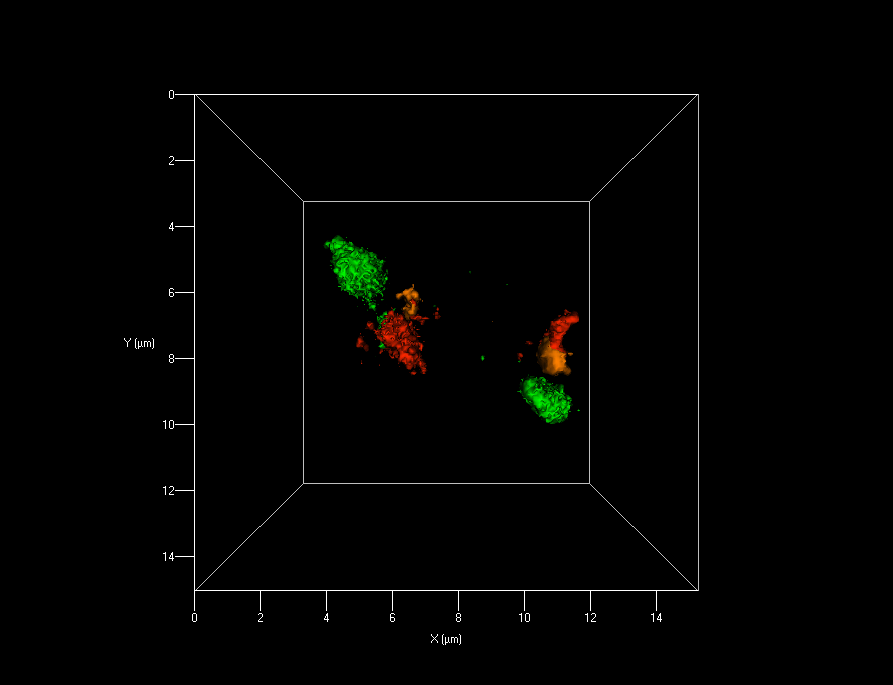

Supplement: Additional file 7 — 3D view of a Pre B nucleus with the orientation pattern “TP”. One chromosome 11 is oriented with its centromeric end (red) towards the nuclear center and with its telomeric end (green) towards the periphery, the other chromosome is located in parallel to the nuclear periphery. [file 1471-2121-15-22-S7.tiff]

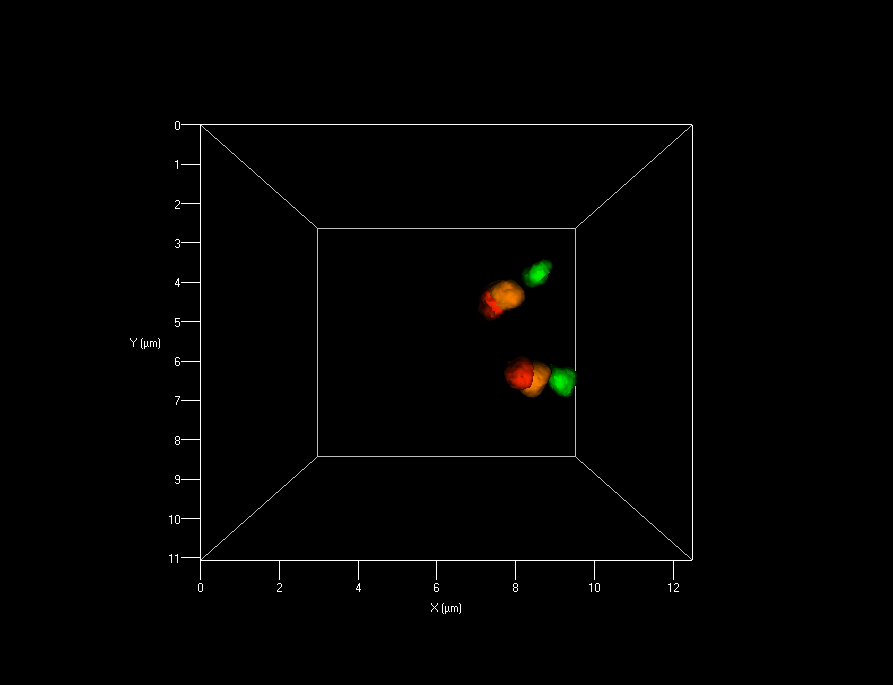

Supplement: Additional file 9 — 3D view of a Pre B nucleus with the orientation pattern “TT”. Both copies of chromosome 11 are pointing with their centromeric ends (red) towards the nuclear center, whereas their telomeric ends (green) are pointing towards the periphery. [file 1471-2121-15-22-S9.tiff]

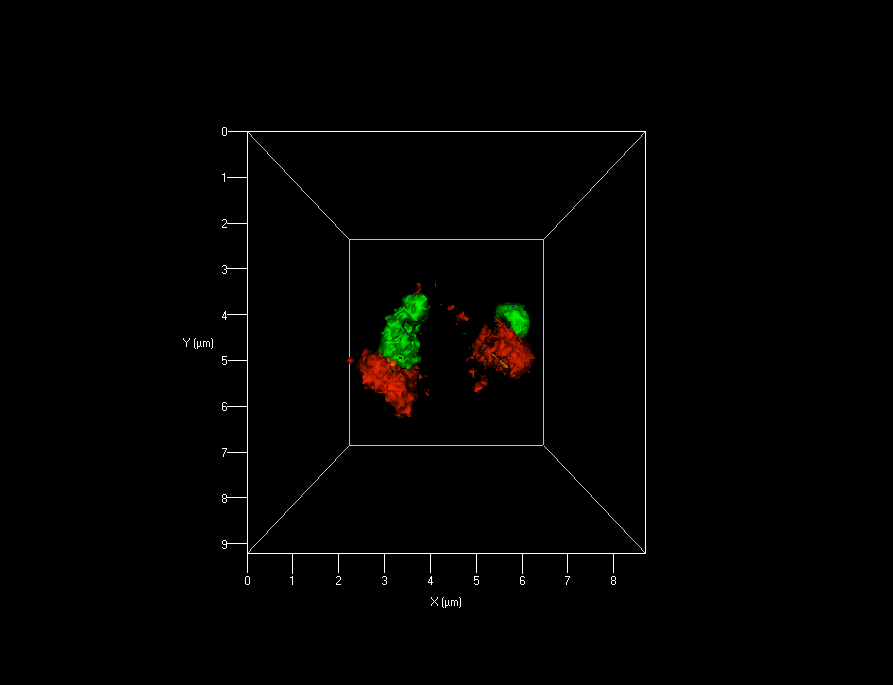

Supplement: Additional file 10 — 3D view of a [T38HxBALB/c]N wild type B cell nucleus with the orientation pattern “CP”. One chromosome 11 is located in parallel to the nuclear periphery, the other is oriented with its telomeric end (green) towards the center. [file 1471-2121-15-22-S10.tiff]

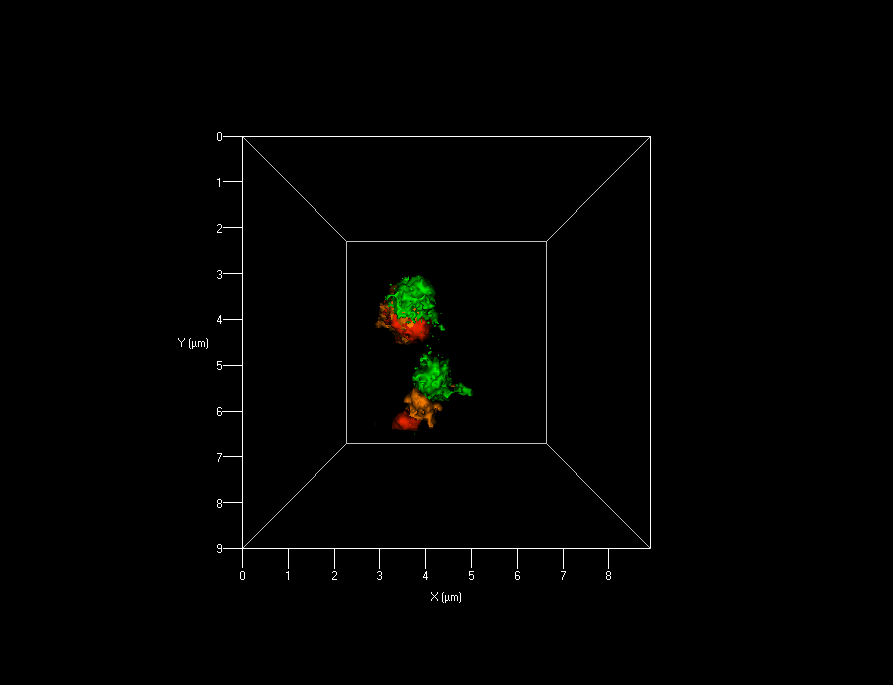

Supplement: Additional file 12 — 3D view of a [T38HxBALB/c]N wild type B cell nucleus with the orientation pattern “CC”. Both copies of chromosome 11 are pointing with their telomeric ends (green) towards the nuclear center. [file 1471-2121-15-22-S12.tiff]

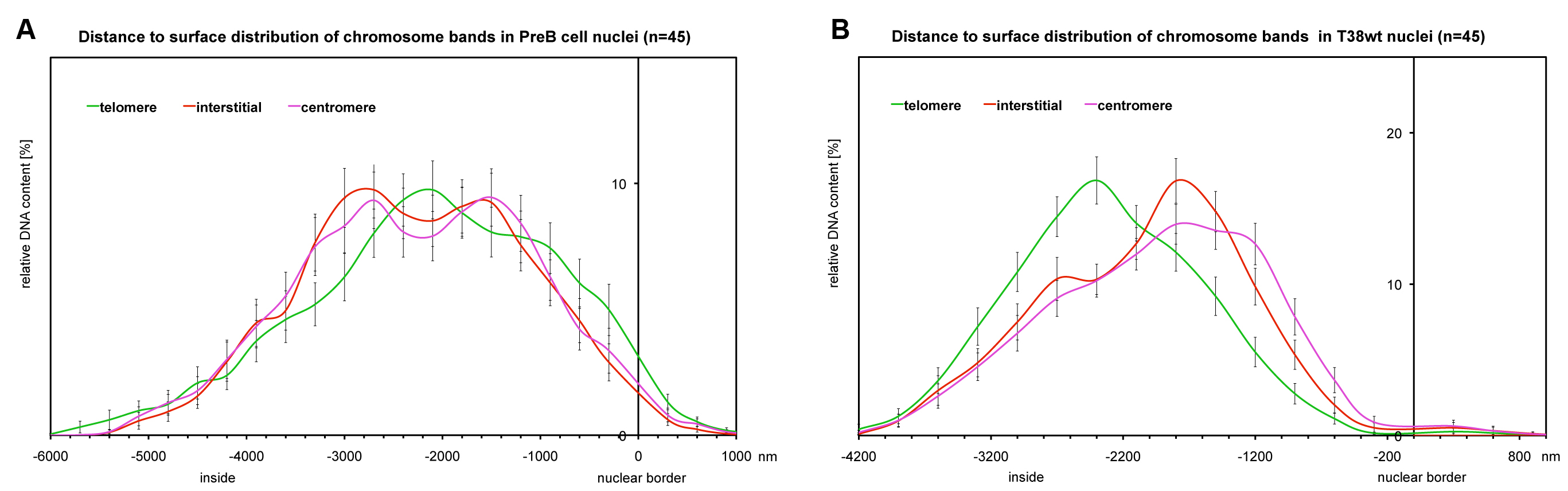

Supplement: Additional file 13 — Mean radial distribution of the centromeric, interstitial and telomeric mBAND FISH signals in A PreB and B T38wt cell nuclei. The 3D FISH signal distance to the nuclear surface was measured nm using eADS software (Küpper et al., 2007) (n = number of nuclei, nm = nanometer). A In PreB cells the mean radial position of the telomeric segment was at 2016 nm and 42% relative distance from the nuclear surface, the interstitial segment at 2178 nm (46%) and the centromeric segment at 2151 nm (45%). B In T38wt cells mean absolute and relative probe distances to nuclear surface were 1906 nm (56%) for the telomeric region, 1617 nm (47%) for the interstitial and 1522 nm (44%) for the centromeric region. A small percentage of nuclei shows band signals beyond the nuclear border. This is probably due to weak nuclear DAPI stain measured by the software eADS (see Discussion). [file 1471-2121-15-22-S13.jpeg]

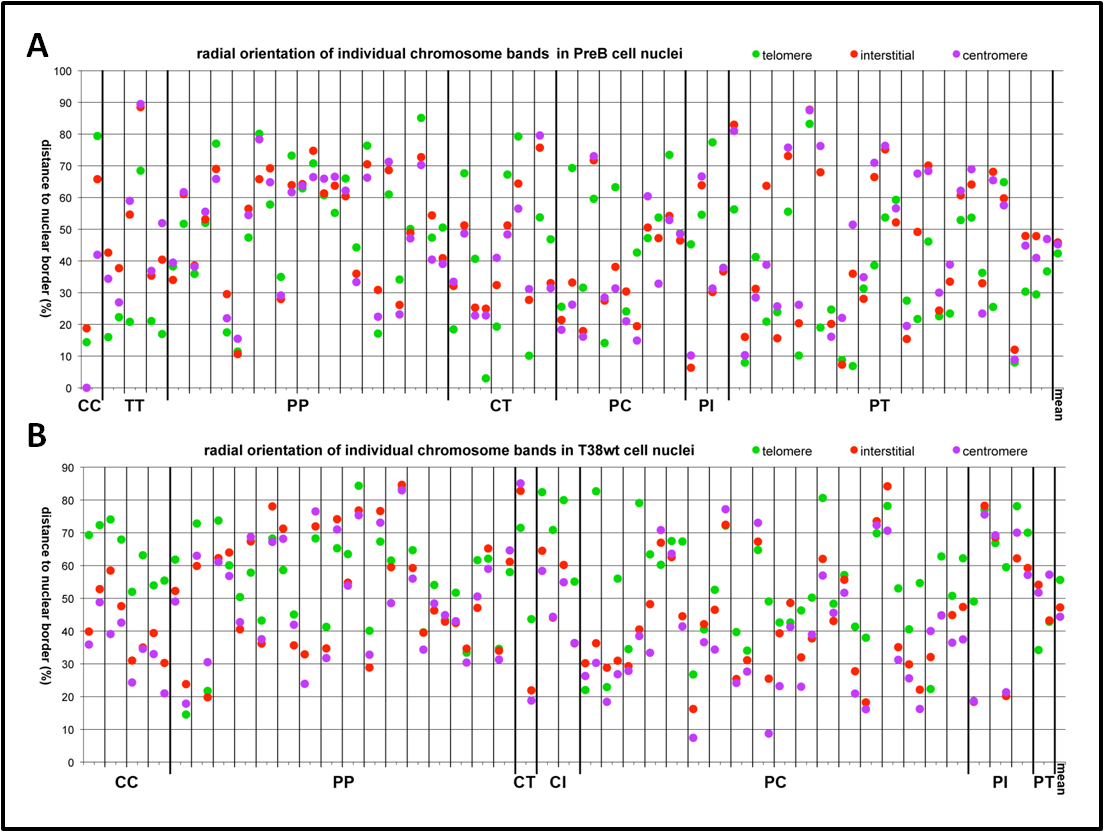

Supplement: Additional file 14 — Normalized (% distance to nuclear surface) radial centromeric, interstitial and telomeric mBAND probe distributions in individual chromosomes from each 45 A PreB and B [T38HxBALB/c]N wild-type B cell nuclei using eADS software. The 3D FISH signal distance to the nuclear surface in nm was transformed to relative values (%) by normalization using the nuclear radius as reference. Each colored dot represents the radial position of the geometric center from an individual chromosome 11. The two chromosome 11 homologs from each nucleus are shown side by side. Nuclei with a similar radial orientation of the two homologs are depicted in clusters separated by bold vertical lines. mBAND territories were designated as parallel (“P”) to the nuclear surface when each of the measured relative radial distances between centromeric, telomeric and interstitial chromosome segments was less than 15% of the nuclear radius. This equals to approximately a 400-500 nm radial distance depending on the size of the respective nucleus. We chose a 15% cut-off level because this is approximately twice the distance between consecutive image z-sections of 200 nm. Consequently, in chromosome 11 territories where at least one of the measured relative distances between mBAND territories would exceed 15% the CT was assigned an orientation with either the telomeric or the interstitial or the centromeric end pointing towards the nuclear periphery or center. (P = parallel, C = centromere points to periphery, I = interstitial is most peripheric, T = telomere points to periphery). [file 1471-2121-15-22-S14.tiff]

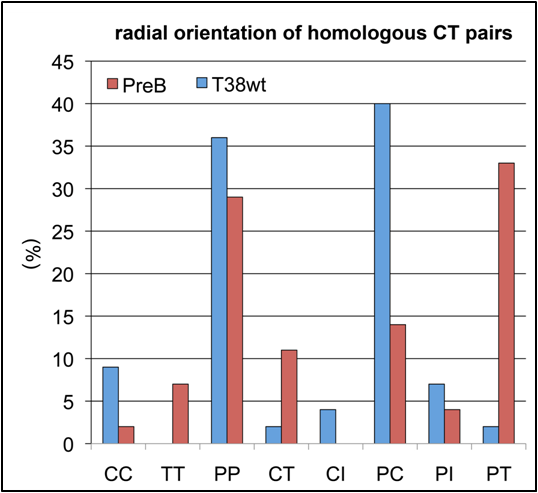

Supplement: Additional file 15 — Relative radial orientation of 45 homologous chromosome 11 mBAND CT pairs from each 45 PreB and [T38HxBALB/c]N wildtype B cell interphase nuclei. Frequencies (%) of homologous CT pairs showing different combinations of radial orientations. (P = parallel, C = centromere points to periphery, I = interstitial is most peripheric, T = telomere points to periphery). [file 1471-2121-15-22-S15.tiff]
