# Supplementary material for: Distinct nuclear orientation patterns for mouse chromosome 11 in normal B lymphocytes
Source: BMC Cell Biol. 2014 Jun 12;15:22. doi: 10.1186/1471-2121-15-22 (PMC4078936; doi:10.1186/1471-2121-15-22)
Supplement: Additional file 16: Table S1 — Results of semi-automated quantitative analysis using the software eADS. Frequencies of relative orientation patterns of homologous chromosomes in individual nuclei. (P=parallel, C=centromere points to periphery, I=interstitial is most peripheric, T=telomere points to periphery). [file 1471-2121-15-22-S16.docx]

**Supplemental Table 1**

|  | **PreB** | **[T38Hx BALB/c]N wt** | **Chi-Square** |
| --- | --- | --- | --- |
| **Both homologs in parallel to the nuclear border**  **(PP)** | 13 (28.9%) | 16 (35.6%) | 0.50 |
| **One homolog points with its centromere to the nuclear periphery, the other is parallel to the nuclear border (CP)** | 6 (13.3%) | 18 (40%) | 0.0042 |
| **One copy points with its telomere to the nuclear periphery, the other is parallel to the nuclear border**  **(TP)** | 15 (33.4%) | 1 (2.2%) | <0.0001 |
| **Both homologs point with their telomeric end to the nuclear periphery (TT)** | 3 (6.7%) | 0 (0%) | 0.08 |
| **One copy points with its telomeric end, and the other copy with centromeric end, to nuclear periphery (CT)** | 5 (11.1%) | 1 (2.2%) | 0.09 |
| **Both copies point with their centromeric ends to the nuclear periphery (CC)** | 1 (2.2%) | 4 (8.9%) | 0.17 |
| **One homolog points with its centromere to the nuclear periphery, the other is positioned with its interstitial region most peripherical (CI)** | 0 (0%) | 2 (4.4%) | 0.15 |
| **One homolog is parallel to the nuclear border, the other is positioned with its interstitial region most peripherical (PI)** | 2 (4.4%) | 3 (6.7%) | 0.65 |
| **TOTAL** | 45 | 45 |  |
